# Trace Metals in Amazonian Rivers Sediments from Northern Brazil: Spatial-Temporal Variations, Sources, and Ecological Risks

**DOI:** 10.3390/toxics13100891

**Published:** 2025-10-18

**Authors:** Larissa Costa de Souza, Camila Carneiro dos Santos, Neuton Trindade Vasconcelos Júnior, Kelson do Carmo Freitas Faial, José Augusto Martins Corrêa, Rosivaldo de Alcântara Mendes

**Affiliations:** 1 Universidade Federal do Pará, Instituto de Geociências, Campus Universitário do Guamá, s/n, Belém 66075-110, PA, Brazil; santos.camilac@gmail.com (C.C.d.S.);; 2 Instituto Evandro Chagas, Seção de Meio Ambiente, Laboratório de Toxicologia, Rodovia BR 316 km 07, Levilândia, Ananindeua 67010-000, PA, Brazil; neutonjunior@iec.gov.br (N.T.V.J.); kelsonfaial@iec.gov.br (K.d.C.F.F.); rosivaldomendes@iec.gov.br (R.d.A.M.)

**Keywords:** trace metals, river pollution, Amazon region, surface sediments

## Abstract

Trace metal pollution has become an increasing concern in urban areas, mainly due to industrial activities and heightened human activities near water bodies. This study aimed to quantify the level of pollution caused by the trace metals Co, Cr, Cu, Mn, Ni, and Zn in surface sediments of Aurá and Guamá rivers, as well as Guajará Bay, in the metropolitan region of Belém (Northern Brazil). A total of 33 sediment samples were collected—14 from the Aurá River, 7 from the Guamá River, and 12 from Guajará Bay—during both the wet and dry seasons to capture seasonal variability. The studied trace metals were measured through inductively coupled plasma optical emission spectrometry (ICP-OES), and the decreasing order of concentration detected was the following: Mn > Zn > Cr > Ni > Co > Cu. To assess the degree of pollution, three geochemical indicators were employed: the Geoaccumulation Index (Igeo), which compares observed concentrations with natural background levels to classify contamination severity; the Enrichment Factor (EF), which helps distinguish between natural and anthropogenic sources of metals using a reference element (typically aluminum or iron); and the Mean-ERM-Quotient, which evaluates the potential ecotoxicological risk of the metals based on benchmark values for adverse effects on aquatic organisms. Based on these indicators, the sediments of the studied area can be classified as showing “moderate contamination and enrichment”. The metals Zn and Cu exhibited the highest degrees of enrichment, likely of anthropogenic origin. Overall, this study revealed that areas closer to sites of intense human activity are more susceptible to trace metal contamination, especially during the wet season. Frequent monitoring of areas classified as “contaminated” and time-series data are necessary to examine more deeply the pollution of river sediments and their potential changes concerning shifts in the status of urbanization and industrialization.

## 1. Introduction

Metals are chemical elements widely present in the Earth’s crust and in various anthropogenic products. Their presence in the environment is an increasing concern due to their specific characteristics, such as non-degradability, high toxicity potential, persistence, and especially their capacity for bioaccumulation and biomagnification. These properties pose a significant threat to ecosystems and human health [1,2,3,4,5,6].

Although metals such as copper (Cu), zinc (Zn), cobalt (Co), manganese (Mn), chromium (Cr), and nickel (Ni) are essential for maintaining vital biological functions, their presence at elevated concentrations can cause negative impacts. These impacts interfere with cellular functions, alter molecular and tissue structures, and compromise the overall functioning of organisms. Moreover, these toxic effects can be exacerbated by the bioaccumulation capacity of metals in aquatic organisms, and consequently, within the food chain, leading to accumulation in the tissues of living beings even in the absence of direct exposure [7,8].

The sedimentary compartment, in turn, is an environmentally significant matrix of heterogeneous nature [9]. Sediments found on the beds of water bodies are formed by the deposition of organic and inorganic particles that settle at the bottom of these environments [10]. This compartment plays a crucial role in aquatic ecosystems, influencing biogeochemical cycles and reflecting the overall quality of the ecosystem [11]. Furthermore, sediments may act not only as sinks and transporters of pollutants but also as secondary sources of contaminants to the environment. Some pollutants can be mobilized into the water column through physicochemical, biological, and even anthropogenic processes [12].

Sediment contamination by metals occurs when the concentration from natural and anthropogenic sources exceeds tolerable limits for ecosystems. Inappropriate disposal of industrial effluents, excessive application of agrochemicals in agricultural practices, and mining and smelting activities are among the main sources that have intensified environmental pollution, especially in urban and industrial regions [7,8].

The Metropolitan Region of Belém (MRB) is traversed by a complex hydrographic system that includes rivers, canals, streams, and artificial lakes of various sizes. The Guamá and Aurá rivers, together with the Guajará Bay, form an interconnected aquatic ecosystem with a hydrodynamic regime characterized by variations in water level and flow velocity [13,14]. This ecosystem faces continuous environmental pressures due to high population density and industrial development—resulting in the release of industrial and domestic effluents—and the unregulated occupation of areas adjacent to conservation zones. These factors contribute to the pollution and contamination of surface water bodies and bottom sediments [15,16]. Therefore, monitoring metal concentrations in sediments is essential to assess pollution extent and maintain environmental quality [17].

In this context, the present study aims to (i) investigate the concentration of trace metals (Co, Cr, Cu, Mn, Ni, and Zn) in sediments from the Aurá and Guamá rivers and Guajará Bay, located in the Metropolitan Region of Belém (MRB), Northern Brazil; (ii) evaluate the potential ecological risks associated with these metals; and (iii) identify the sources and contexts of pollution through the application of multivariate analyses.

## 2. Materials and Methods

### 2.1. Study Area and Sampling Site

The city of Belém and its metropolitan region have an estimated population of approximately 2.27 million inhabitants. The area is located at an average altitude of 15 m above sea level, at the confluence of the Guamá River (to the south) and the Guajará Bay, influenced by both marine and fluvial factors. The water supply network reaches about 80% of households, but only 8% of household discharges are connected to the sewage collection system, resulting in the discharge of untreated waste [18].

The Aurá River, located between the cities of Belém and Ananindeua (State of Pará, Northern Brazil), exerts a significant influence on water catchment sources, supplying around 75% of Belém’s population, since the catchment point is near the river’s mouth. Main environmental issues include deforestation, flooding, erosion, water contamination, and the continuous input of chemical compounds from the illegal landfill, despite its official deactivation since 2015 [19].

The Guamá River supplies water to the Utinga water complex (comprising the Água Preta and Bolonha lakes), which is treated and distributed to the population [20,21]. Beyond public water supply, it is vital for fishing, navigation, recreation, industry, tourism, and commerce [14]. However, unplanned urban growth and sanitation infrastructure increase surface runoff and transport of organic and inorganic materials, leading to degradation of this water resource [22].

The Guajará Bay, located on the right bank of the Pará River, receives inputs from the Guamá and Acará rivers, and is influenced by tides due to its proximity to the Atlantic Ocean [23,24,25]. It also experiences intense cargo movement, vessel fueling, illegal sewage dumping, and other port activities [16].

### 2.2. Sediment Sampling and Pretreatment

**Figure 1:**
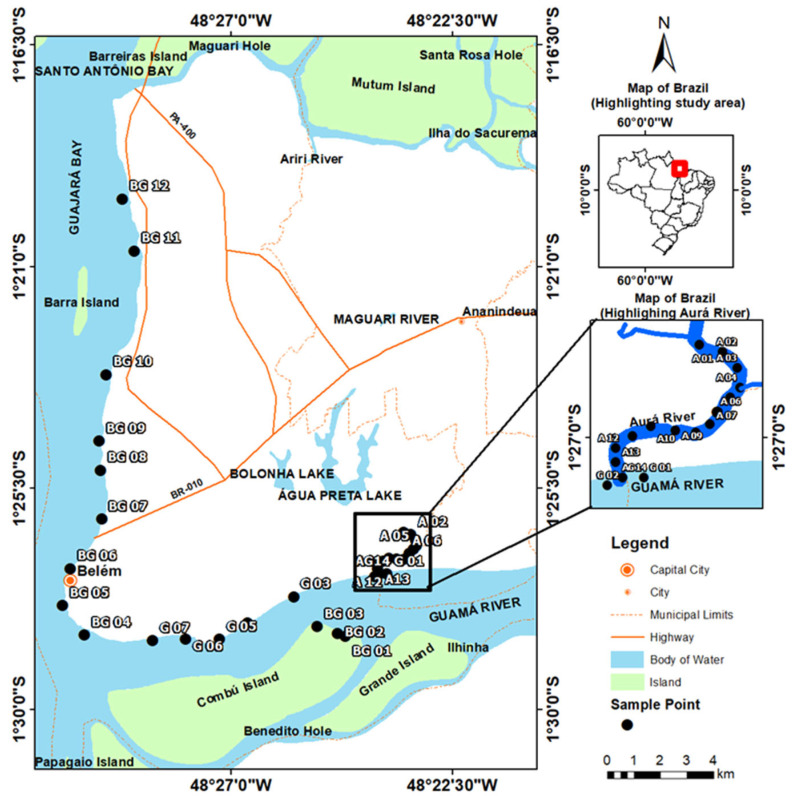
Location of the study area (Aurá and Guamá rivers, and Guajará Bay, in the Metropolitan Region of Belém-PA) with identification of the sampling points.

Surface sediments were collected during the dry season (September 2022) and wet season (April 2023), always during ebb tide, at 14 sampling points along the Aurá River (A 01–A 14), 07 along the Guamá River (G 01–G 07), and 12 in the Guajará Bay (BG 01–BG 12) (Figure 1).

The sediment sampling methodology followed the recommendations of Loring and Rantala (1992) [26], utilizing a Van Veen grab sampler. All sediment samples were stored in plastic bags (free of Bisphenol A), labeled, and refrigerated until transfer to the Organic Residue Analysis Laboratory (LARO) at the Evandro Chagas Institute (IEC).

Samples were air-dried at room temperature and then disaggregated using a mortar and pestle. Approximately 50% of each sample was sieved through a 270-mesh screen and stored in vials for later analysis. The remaining portion was reserved for organic carbon determination and granulometric analysis.

### 2.3. Sediment Characterization

The pH of the samples was measured using a benchtop multiparameter device (Seven2go—METTLER TOLEDO). Organic carbon (COrg) content was determined gravimetrically (450 °C for 4 h) through oxidation of organic matter. Granulometry was performed using a laser particle analyzer (Laser Diffraction SALD 2101—SHIMADZU), and sediment texture was classified based on the relative proportions of clay (<4 µm), silt (4–63 µm), and sand (>63 µm) [27].

### 2.4. Analysis of Trace Metals

Approximately 0.2 g of each sediment sample was digested following the USEPA 3050B method: acidification with 3 mL of nitric acid and 1 mL of hydrochloric acid, followed by microwave radiation (MARSXPRES, CEM) at 1500 W and 175 °C for 10 min.

The qualitative and quantitative analysis of the six evaluated trace metals (Co, Cr, Cu, Mn, Ni, and Zn) was performed using inductively coupled plasma optical emission spectrometry (ICP-OES) (Vista MPX CCD Simultaneous—VARIAN). Wavelengths used for each analyte were: 230.786 nm (Co), 267.716 nm (Cr), 327.395 nm (Cu), 257.610 nm (Mn), 231.604 nm (Ni), and 213.857 nm (Zn). Operating conditions for ICP-OES are summarized in Table 1.

Quality control involved the use of certified reference materials (CRM 2710 and CRM 2711). Recovery rates ranged from 78% to 97% across all elements.

### 2.5. Assessment of Sedimentary Contamination Levels

To evaluate metal contamination in sediments and the influence of anthropogenic activities, geochemical indices were employed [28,29]. Background concentrations were based on average crustal levels [30], and the concentration of Aluminum (Al) was used for normalization, given its abundance and conservative behavior in sediments [31].

#### 2.5.1. Geoaccumulation Index (Igeo)

Initially proposed by Muller (1969) [32], Igeo assesses contamination levels and was calculated using the formula (Equation (1)) adapted from Xu et al. (2014) [33].

(1) $$\mathrm{Igeo}=\log_{2}\left(\frac{\left(\frac{M}{Al}\right)Sample}{1.5*\left(\frac{M}{Al}\right)Background}\right)$$

where *M sample* = concentration of the metal in the sediment sample; *Al sample* = aluminum concentration in the sample; *M background* = background or average crustal value of the metal; *Al background* = background or average crustal aluminum value; 1.5 = correction factor accounting for lithogenic variations and to detect minor anthropogenic influence.

Classification of Igeo follows Müller’s (1969) [32] scheme [33]: Igeo ≤ 0 (Class 0—practically uncontaminated); 0 < Igeo < 1 (Class 1—uncontaminated to moderately contaminated); 1 < Igeo < 2 (Class 2—moderately contaminated); 2 < Igeo < 3 (Class 3—moderately to heavily contaminated); 3 < Igeo < 4 (Class 4—heavily contaminated); 4 < Igeo < 5 (Class 5—heavily to extremely contaminated); 5 ≥ Igeo (Class 6—extremely contaminated).

#### 2.5.2. Enrichment Factor (EF)

The Enrichment Factor (EF) is a useful tool for determining the degree of anthropogenic pollution caused by trace metals [28] and is calculated according to Equation (2):

(2) $$\mathrm{EF}\;=\;\left[\frac{\left(\frac{M}{Al}\right)Sample}{\left(\frac{M}{Al}\right)\;Background}\right]$$

where *M sample* = concentration of the metal in the sediment sample; *Al sample* = concentration of aluminum in the sample; *M background* = background or average crustal value of the metal; *Al background* = background or average crustal aluminum value.

According to Araújo and Souza (2012) [34], environmental contamination indicated by EF can be classified as follows: Enrichment Deficiency (EF < 2), Moderate Enrichment (2 < EF < 5), Significant Enrichment (5 < EF < 20), Very High Enrichment (20 < EF < 40); and Extremely High Enrichment (EF > 40).

#### 2.5.3. Mean-ERM-Quotient (M-ERM-Q)

The Mean-ERM-Quotient (M-ERM-Q) is a beneficial tool for condensing large amounts of pollutant data into a single value. This index can be used to identify and prioritize areas of potential hazards related to sediment quality [35,36]. M-ERM-Q is calculated according to Equation (3) [37]:

(3) $$M-ERM-Q=\;\frac{\sum Ci/ERMi}{n}$$

where Ci = concentration of element (i) in the sediments; ERMi (Median Range Effects) = guideline values reported by Long et al. (1995) [38] for trace metals; *n* = number of trace metals considered.

For the calculation of the M-ERM-Q, a combination of four trace metals (Cr, Ni, Zn, and Cu) was employed. The classification categories for M-ERM-Q are presented in Table 2.

### 2.6. Statistical Analysis

Descriptive statistical analysis (mean, standard deviation, and coefficient of variation) was employed to evaluate the data and explore trace metal levels and distribution. Principal Component Analysis (PCA) and matrix correlation analyses were performed to investigate the relationships between the variables analyzed and their distributions. PCA is a multivariate technique used to simplify the interpretation of complex systems; its primary function is to reduce the number of variables while preserving as much of the original information as possible [41].

Another statistical tool employed was the Spearman–Pearson correlation analysis, which is widely used in environmental studies due to its efficiency in revealing relationships between multiple variables. Correlation matrices included pH, organic carbon (COrg), grain size, and trace metal concentrations, and were generated separately for the dry and wet seasons. Statistical analyses were performed with Origin 2024 software.

## 3. Results

### 3.1. Sediment Characterization

Geochemical characteristics, such as COrg content and grain size, showed minor variations, reflecting a specific distribution pattern. During the dry season, sediment samples were dominated by the silt fraction (average of 66.32% ± 10.36), attributed to lower hydrodynamic activity in the rivers, which results in greater sedimentation of fine particles near the river banks, the area where sampling was conducted. In wet season, the sand fraction predominated (average of 59.28% ± 13.45), due to increased rainfall that enhances particulate matter transport into the river and intensifies water turbulence. This turbulence causes sediments with lower grain size (silt + clay) to remain in suspension, while larger particles with a higher grain size settle.

The COrg content in the sediment samples varied seasonally, ranging from 4.0 to 13.0% during the dry season and 2.0 to 10.0% during the wet season. This pattern is justified by the affinity of COrg for the fine fraction (% Fines), which was also higher during the dry season when reduced hydrodynamics favored sedimentation. It is also because the reduction in turbulent water movement during this period reduces sediment oxygenation, slowing down the degradation process of organic compounds and favoring their preservation in the sedimentary matrix. During the dry season, primarily, most of the sampling sites had COrg > 5.8%, indicating that the sediment is predominantly organic and suggesting an accumulation of organic matter in the studied area [42,43].

Regarding pH, the sediment samples were characterized as slightly acidic (mean pH = 5.37 ± 0.42) across both study periods and all sampling sites. The least acidic pH was recorded in Guajará Bay (pH = 6.21), while the most acidic was found in the Aurá River (pH = 4.20), both during the dry season. These results were expected due to the organic nature of the sediments, as the decomposition of organic matter (OM) leads to the formation of sulfuric acid, which increases the concentration of H^+^ ions [43], resulting in the observed acidity of the sediments. The lower pH observed in the Aurá River may also be associated with its smaller stream volume and less vigorous hydrodynamic conditions compared to the other aquatic systems investigated.

### 3.2. Spatial and Seasonal Distribution of Trace Metals in Sediments and Sediment Quality Guidelines

Descriptive statistics of the metals evaluated in this study are presented in Table 3. The mean concentrations followed a decreasing order: Mn > Zn > Cr > Ni > Co > Cu. The average concentrations of trace metals were below background levels (Al = 82,120.00 mg kg^−1^, Co = 38.00 mg kg^−1^, Cr = 228.00 mg kg^−1^, Cu = 37.40 mg kg^−1^, Mn = 929.00 mg kg^−1^, Ni = 99.00 mg kg^−1^, Zn = 79.00 mg kg^−1^) [29], and remained below the guideline values (TEL and PEL) established by the Canadian Council of Ministers of Environment, which are also adopted by the Environmental Technology and Sanitation Company of the State of São Paulo (CETESB). The TEL (Threshold Effect Level) indicates the concentration below which adverse effects on organisms are rarely expected, while the PEL (Probable Effect Level) indicates the concentration above which adverse effects are more likely to occur [44].

The concentration ranges of trace metals (in mg kg^−1^) during the dry season were as follows: 0.35–12.78 for Co, 5.55–14.22 for Cr, 1.80–30.23 for Cu, 68.64–501.72 for Mn, 0.05–23.82 for Ni, and 19.68–102.87 for Zn. During the wet season, concentrations were as follows: 0.06–12.91 for Co, 7.71–24.52 for Cr, 3.44–29.29 for Cu, 175.03–557.73 for Mn, 0.05–29.09 for Ni, and 21.06–124.41 for Zn. Among the metals evaluated, Zn, Mn, and Cu showed the greatest variations and levels, accounting for more than 90% of the total concentration of the metals quantified.

**Figure 2:**
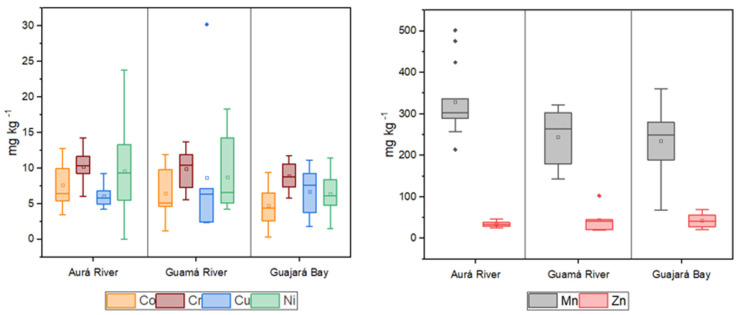
Distribution of trace metals within the study area during the dry season.

**Figure 3:**
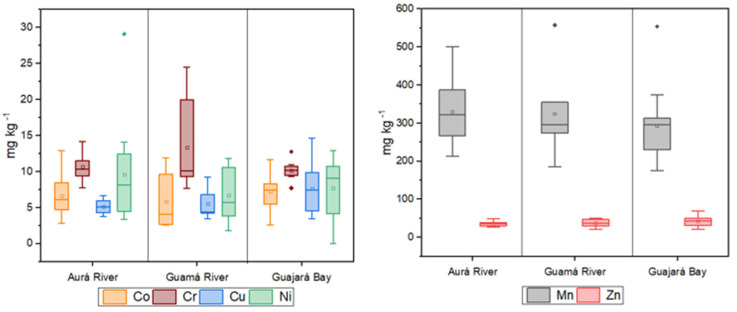
Distribution of trace metals within the study area during the wet season.

According to the classification of trace metals variability by de Abuduwaili et al. (2015) [45] and Lu et al. (2012) [46], the variations in Co, Cr, Cu, Mn, Ni, and Zn were 46.29%, 26.98%, 59.04%, 31.29%, 62.28%, and 35.45%, respectively. These results indicate moderate variability, suggesting a heterogeneous distribution. Figure 2 and Figure 3 illustrate the spatial distribution of the analyzed metals within the study area during the dry and wet seasons, respectively.

Metal concentrations analysis revealed significant variations in contamination levels. Co concentrations were higher in the Aurá River during both the dry season (A 03) and the wet season (A 10), with the highest levels observed during the wet season. This may be associated with sediment leaching processes or the presence of punctual sources of contamination near these sampling sites, which could be more evident during periods of higher water flow.

Cr and Mn displayed distinct patterns: higher levels were observed during the dry season in the Aurá River at site A 04 (Cr) and site A 11 (Mn), while during the wet season, elevated levels were detected in the Guamá River at site G 04 (Cr) and site G 05 (Mn). These variations may be influenced by climatic factors such as increased surface runoff during the wet seasons, which can transport contaminated material from farther areas, thereby increasing metal concentration. Additionally, natural characteristics of the rivers, such as organic matter content and sediment types, may affect the retention and mobilization of these metals.

Mn concentrations observed were lower than those reported by He et al. (2023) [47] in the Brisbane River, Australia, and comparable to levels found by Das et al. (2023) [48] in the Ganges River, India, and by Kang et al. (2023) [49] in the Xihe River, northeastern China. In these studies, certain detected values were identified as potentially concerning. Elevated Mn levels, particularly in sedimentary environments, can disrupt biochemical and biogeochemical processes and may adversely affect sensitive aquatic species [47].

In contrast, Ni was the only studied metal whose concentrations exceeded the limits established by CONAMA resolution No. 454/2012, which concerns dredge material. The highest Ni levels were detected in the Aurá River during both dry (A 04) and wet seasons (A 13). The fact that Ni levels surpass permitted limits indicates a significant contamination source at these sites, warranting further investigation. Ni is widely used in industrial activities and can be toxic to aquatic life. Ni concentrations detected were higher than those reported by Afzaal et al. (2022) [50] in the Kabul River, Pakistan, and comparable to levels found by Tian et al. (2020) [51] in the Bohai and Yellow Seas, northeastern China, and Silva (2021) [52] in the Pelotas River basin.

Cu and Zn levels were highest in the Guamá River, at site G 07, during both sampling periods. This site exhibited significantly elevated levels compared to the other locations, likely due to its proximity to the waterway terminal. Human activities related to cargo handling and vessel operations are probable contributors to increased metal levels, given their association with industrial and maritime processes. Cu concentrations were similar to those reported by Kawichai et al. (2023) [53] in the Mae Chaem River, Thailand, Hasan et al. (2023) [54] in the Pasur River, Bangladesh, and Tureck et al. (2024) [55] in Babitonga Bay, Santa Catarina. Zn levels were comparable to those found by Xiao et al. (2021) [56] in the Lijiang River, China, Siddique et al. (2021) [57] in the Meghna River, Bangladesh, and Bernal et al. (2024) [58] in the Almendares River, Cuba.

### 3.3. Sediment Contamination Assessment

#### 3.3.1. Geoaccumulation Index (Igeo)

**Figure 4:**
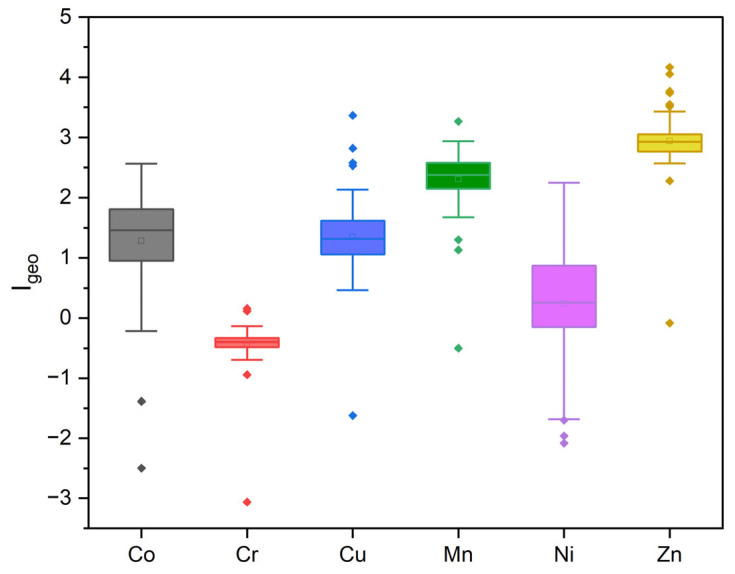
Boxplots of the Geoaccumulation Index (Igeo) for the evaluated trace metals in surface sediments from the study area.

The Igeo values for the evaluated trace metals (except Cr) were positive (IGeo ≥ 0) across the studied area. During the dry period, the average Igeo values ranged from −2.49 to 2.48 for Co, 0.46 to 3.36 for Cu, 1.13 to 3.26 for Mn, −1.96 to 2.14 for Ni, and 0.12 to 4.16 for Zn. During the wet period, the ranges were from −1.38 to 2.56 for Co, −1.61 to 2.57 for Cu, −0.50 to 2.93 for Mn, −2.08 to 2.25 for Ni, and −0.08 to 3.76 for Zn. Based on this index, the sediments were classified as moderately to heavily contaminated, with 0.0 < Igeo < 3.50 (Figure 4).

#### 3.3.2. Enrichment Factor (EF)

**Figure 5:**
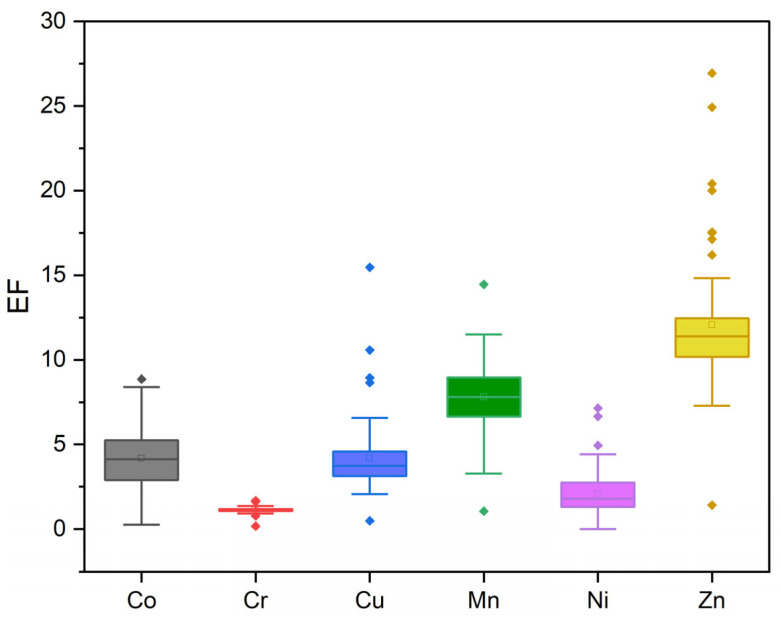
Boxplots of the Enrichment Factor (EF) for the evaluated trace metals in surface sediments from the studied area.

The EF used to assess the degree of anthropogenic pollution by the studied trace metals is presented in Figure 5. For both seasons, the samples exhibited EF values ranging from 0 to 27 for Co, Cr, Cu, Mn, Ni, and Zn, indicating enrichment with these metals. Cr showed the lowest enrichment, with an EF between 0.17 and 1.67. Co (0.45 < EF < 8.85), Cu (0.48 < EF < 15.46), Mn (1.06 < EF < 14.45), and Ni (0.0 < EF < 7.14) displayed significant enrichment, while Zn (1.41 < EF < 6.93) showed very high enrichment.

The highest EF values for Cu, Mn, and Zn were observed in the samples from the dry season, potentially related to lower hydrodynamic activity and higher COrg during this period. Overall, the contamination levels indicated by EF were consistent with the Igeo classification, suggesting accumulation of trace metals—particularly Zn and Mn—in the sediments of the region. These metals may originate from natural sedimentary processes related to the Barreiras Formation and the Earth’s crust, especially from sedimentary rocks [59], as well as from anthropogenic activities such as domestic sewage discharge [60,61,62].

#### 3.3.3. Mean-ERM-Quotient (M-ERM-Q)

**Figure 6:**
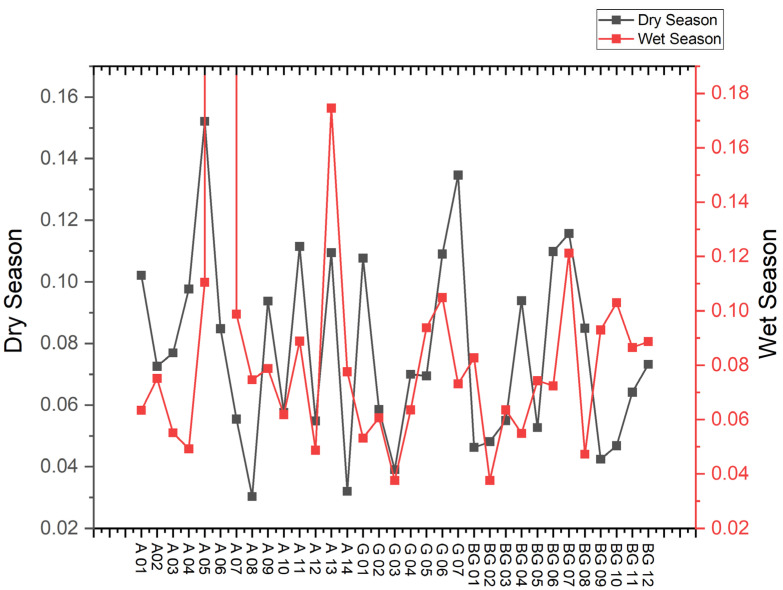
Spatial and seasonal variations of Mean-ERM-Quotient (M-ERM-Q) for trace elements (n = 4) in sediments from the studied area.

The samples predominantly exhibited M-ERM-Q values ≤ 0.1 (Figure 6), classifying the study area as a “low priority” for potential toxicity. The minimum value (M-ERM-Q = 0.030) was found in dry season samples, while the maximum value (M-ERM-Q = 0.175) occurred during the wet season, both in the Aurá River. The sediments indicated a 9% probability of toxicity, suggesting a low potential risk to aquatic organisms.

### 3.4. Statistical Analysis

Spearman correlation analysis was performed to evaluate relationships between sediment geochemical properties and trace metals during both monitoring periods. Data highlighted in bold in Table 4 and Table 5 indicate statistically significant correlations (*p* ≤ 0.05).

During the dry season, several significant correlations were observed among trace metals, Corg, pH, and sediment grain size fractions. Notably, a strong positive correlation was identified between Cr and Corg (r = 0.74), suggesting a potential association of Cr with organic matter in the sediments. Additionally, significant correlations were observed between Cr and Zn (r = 0.58), Cr and Mn (r = 0.58), and Cu and Zn (r = 0.93). These findings imply the possibility of co-occurrence or a common anthropogenic source for these elements. The results indicate that during the dry season, trace metals tend to exhibit stronger associations with each other and with organic matter, potentially reflecting immobilization within the sediment matrix.

In contrast, during the wet season, the correlation patterns differed markedly, with fewer significant relationships detected among trace metals and sediment characteristics. Nonetheless, some notable associations persisted; the strongest was between Cu and Zn (r = 0.88), followed by Cr and Zn (r = 0.71), and Cr and Mn (r = 0.67). These correlations suggest that these elements maintain coupled behavior despite the increased hydrosedimentary dynamics characteristic of the wet season. Conversely, no significant correlations were observed between trace metals and either Corg or pH during this period, indicating that enhanced mobilization of particles may diminish the influence of organic matter on metal retention.

According to Sanches and Peil (2015) [63] and Lemes et al. (2003) [64], sediments dominated by fine fractions (<63 μm) have a greater capacity to retain metals. As the grain size decreases, the concentrations of nutrients and contaminants tend to increase in bottom sediments, which generally contain higher metal levels compared to coarser fractions, reflecting an increase in reactive surface area, adsorption potential, and coprecipitation [65,66]. Nonetheless, the results indicated that the distribution of trace metals in the studied sediments is influenced by factors unrelated to grain size.

The transport and spatial distribution of trace metals exhibit seasonal variability, driven by multiple inputs from both point and diffuse pollution sources. Significant intermetallic correlations suggest that trace metals share common sources, exhibit interconnected behaviors, and undergo similar transport mechanisms from sources to sinks [36,67,68]. The most significant factor in the heterogeneous distribution pattern of metals in the studied area appears to be local hydrodynamics.

**Figure 7:**
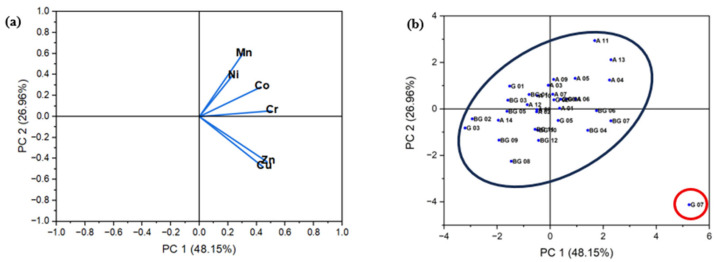
PCA analysis from dry season. (**a**) Factor loadings of six trace metals on PC 1 (48.15%) and PC 2 (26.96%). (**b**) Factor scores of sampling sites and their influence zones. The red group is primarily composed of petrogenic trace metals.

Principal Component Analysis (PCA) was employed to identify patterns in the trace metal distribution and potential contamination sources during dry and wet seasons. Figure 7 andFigure 8 present the factor loadings (a) and sampling sites scores (b) corresponding to the first two principal components for each seasonal period.

In the dry season (Figure 7), the first two components accounted for 75.11% of the total variance, with 48.15% explained by the first component (PC1) and 26.96% by the second (PC2). The factor loadings (Figure 7a) reveal strong correlations among specific trace metals, indicating a probable common origin. The analysis of the factor scores (Figure 7b) shows the formation of a distinct cluster of sampling points (in red), which may correspond to areas heavily influenced by anthropogenic activities, such as effluent discharges from domestic, industrial, and urban sources near water bodies.

**Figure 8:**
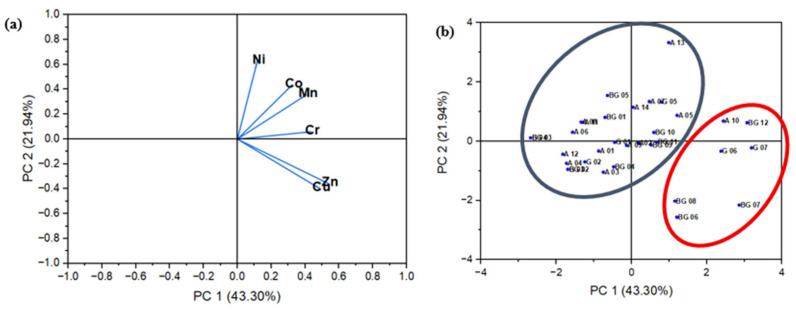
PCA analysis from wet season. (**a**) Factor loadings of six trace metals on PC 1 (43.30%) and PC 2 (21.94%). (**b**) Factor scores of sampling sites and their influence zones. The red group is primarily composed of petrogenic trace metals.

During the wet season (Figure 8), the combined variance explained by the first two components decreased slightly to 65.24%, with PC1 explaining 43.30% and PC2 accounting for 21.94%. This reduction may reflect increased complexity in metal transport dynamics due to intensified surface runoff, leaching, and the mobilization of diffuse pollutants. Nonetheless, the factor loadings (Figure 8a) demonstrate clustering among certain metals, reinforcing the influence of anthropogenic sources. The spatial distribution of sampling points in the score plot (Figure 8b) indicates a redistribution that suggests seasonal processes enhance contaminant dispersion, especially in areas affected by urbanization.

In both considered periods, the observed clustering in the score plots suggests the continued influence of anthropogenic activities contributing to metal contamination, likely associated with ongoing urban development and land use in the study region.

## 4. Conclusions

This study highlights the seasonal distribution of the trace metals Co, Cr, Cu, Mn, Ni, and Zn in sediments within an intensively exploited aquatic ecosystem influenced by commercial and industrial activities. The region’s high population density contributes to pollution through industrial and domestic effluents. In general, factors such as grain size, pH, and organic carbon (Corg) are related to metal behavior in sediments; therefore, they were also taken into account. Among the metals evaluated, Zn, Mn, and Cu showed the greatest variations and levels, accounting for more than 90% of the total concentration of the metals quantified.

Spearman correlation analysis indicated that the distribution of trace metals in sediments is influenced by factors unrelated to grain size; the most significant factor in the heterogeneous distribution pattern of metals appears to be local hydrodynamics. Principal Component Analysis (PCA) enables characterization of the sampling sites based on the first and second components and reveals the presence of two groups of correlated compounds during both seasons, one composed of metals derived from a mixture of sources and the other predominantly influenced by anthropogenic activity. In both seasons, the observed clustering in the score plots suggests the continued influence of anthropogenic activities contributing to metal contamination, mainly associated with ongoing urban development and land use in the study region.

The Geoaccumulation Index (Igeo), Enrichment Factor (EF), and the Mean-ERM-Quotient indicated that the sediments of the studied area can be classified as showing “moderate contamination and enrichment”. Overall, the analysis demonstrated that sites located near areas of intense human activity are more susceptible to trace metal contamination, especially during the wet season. This contamination may negatively impact the aquatic ecosystem and public health if pollution sources are not properly managed.

Although most of the metals analyzed were within limits established by CONAMA, continuous monitoring is essential to prevent contamination levels from exceeding permissible limits, thus avoiding potential environmental and biodiversity damage. Analyzing trace metals in surface sediments provides vital data for ecological management and helps inform policies aimed at protecting and restoring water quality. Further studies are necessary to identify pollution sources and assess the temporal dynamics of these metals, enabling a more detailed understanding of contamination processes and their ecological consequences.

## Figures and Tables

**Table 1:** Operating parameters of the ICP-OES Vista-MPX used for trace metal analysis.

Parameters	Conditions
RF Generator	40 MHz
RF Power	1.0 kw
Plasma Flow	15 L min^−1^
Auxiliary Gas Flow	1.5 L min^−1^
Flow of Nebulization gas	0.7 L min^−1^
Sample Pump Flow	1.0 L min^−1^

**Table 2:** M-ERM-Q classification [36,39,40].

M-ERM-Q Value	Risk Level	Probability of Being Toxic
≤0.1	Low priority	9%
0.1 < M-ERM-Q ≤ 0.5	Medium—Low priority	21%
0.5 < M-ERM-Q ≤ 1.5	High—Medium priority	49%
>1.5	High priority	76%

**Table 3:** Descriptive statistics of trace metals evaluated in surface sediments of the study area.

Metals	Range (mg kg^−1^)	Mean (mg kg^−1^)	SD (mg kg^−1^)	CV (%)	BV (mg kg^−1^)	TEL (mg kg^−1^)	PEL (mg kg^−1^)
Co	0.35–12.91	6.50	3.01	46.29	38.00	-	-
Cr	5.55–24.52	10.64	2.86	26.98	228.00	37.3	90.0
Cu	1.80–30.20	6.49	3.83	59.04	37.40	35.7	197.0
Mn	68.64–557.73	296.37	92.72	31.29	929.00	-	-
Ni	0.046–29.09	8.28	5.16	62.28	99.00	18.0	35.9
Zn	19.68–102.87	38.97	13.82	35.45	79.00	123.1	315.0

SD—Standard Deviation, CV—Variable Coefficient, BV—Background Values, TEL—Threshold Effect Level, PEL—Probable Effect Level.

**Table 4:** Spearman correlation coefficients for parameters (trace metals, pH, organic carbon, and grain size) in sediments from the study area during the dry season.

	Corg	Sand	Silt	Clay	pH	Co	Cr	Cu	Mn	Ni	Zn
Corg	**1**	−0.32	0.29	0.27	0.02	0.29	**0.74**	**0.46**	0.14	0.33	**0.44**
Sand	−0.3	**1**	**−0.91**	−0.69	0.46	−0.43	**−0.48**	−0.26	−0.32	−0.27	−0.17
Silt	0.29	**−0.91**	**1**	0.42	−0.43	0.30	0.39	0.26	0.22	0.18	0.18
Clay	0.25	**−0.69**	0.38	**1**	−0.43	**0.45**	**0.40**	0.06	**0.41**	0.32	−0.01
pH	0.02	0.47	−0.43	−0.33	**1**	−0.24	−0.57	0.01	−0.11	−0.19	0.17
Co	0.35	−0.43	0.30	0.49	−0.23	**1**	**0.60**	0.19	**0.60**	**0.38**	0.22
Cr	**0.74**	**−0.49**	0.39	0.25	−0.05	0.60	**1**	**0.60**	**0.58**	**0.43**	**0.58**
Cu	**0.45**	−0.27	0.26	0.10	0.01	0.19	**0.60**	**1**	0.17	0.28	**0.93**
Mn	0.14	−0.33	0.22	0.52	−0.11	**0.60**	**0.58**	−0.17	**1**	**0.36**	0.15
Ni	0.33	−0.27	0.18	0.25	−0.19	0.38	**0.43**	0.28	0.36	**1**	0.30
Zn	0.44	−0.17	0.18	0.02	0.17	0.22	**0.58**	**0.93**	0.15	0.30	**1**

**Table 5:** Spearman correlation coefficients for parameters (trace metals, pH, organic carbon, and grain size) in sediments from the study area during the wet season.

	Corg	Sand	Silt	Clay	pH	Co	Cr	Cu	Mn	Ni	Zn
Corg	**1**	−0.32	**0.34**	0.21	0.18	0.17	**0.35**	0.02	0.20	0.12	0.21
Sand	−0.32	**1**	**−0.99**	**−0.94**	0.30	0.24	−0.09	−0.05	−0.05	0.11	−0.01
Silt	**0.34**	**−0.99**	**1**	**0.93**	−0.29	−0.25	0.11	0.05	0.07	−0.10	0.01
Clay	0.21	**−0.94**	**0.93**	**1**	−0.26	−0.31	0.09	0.05	0.01	−0.12	−0.01
pH	0.18	0.30	−0.29	−0.27	**1**	0.19	0.21	0.15	0.17	−0.06	0.27
Co	0.16	0.24	−0.25	−0.31	0.19	**1**	0.22	0.18	**0.39**	0.26	0.21
Cr	**0.35**	−0.09	0.11	0.09	0.21	0.22	**1**	**0.63**	**0.67**	**0.42**	**0.71**
Cu	0.02	−0.04	0.04	0.05	0.15	0.18	**0.63**	**1**	0.22	0.11	**0.88**
Mn	0.20	−0.05	0.07	0.01	0.17	**0.39**	**0.67**	0.22	**1**	0.16	0.30
Ni	0.12	0.11	−0.10	−0.12	−0.06	0.26	**0.42**	0.11	0.16	**1**	−0.07
Zn	0.21	0.01	−0.01	−0.01	0.26	0.21	**0.71**	**0.88**	0.30	0.07	**1**

## Data Availability

The authors have stored all the necessary databases for anyone who might be interested in making a query.

## Abbreviations

The following abbreviations are used in this manuscript:

MRB	Metropolitan Region of Belém
COrg	Organic Carbon
LARO	Organic Residue Analysis Laboratory
IEC	Evandro Chagas Institute
USEPA	United States Environmental Protection Agency
IGeo	Geoaccumulation Index
EF	Enrichment Factor
ERM	Median Range Effects
OM	Organic Matter
TEL	Threshold Effect Level
PEL	Probable Effect Level
SD	Standard Deviation
CV	Variable Coefficient
BV	Background Values
PCA	Principal Component Analysis
